# A Novel Small Molecule Which Increases Osteoprotegerin Expression and Protects Against Ovariectomy-Related Bone Loss in Rats

**DOI:** 10.3389/fphar.2019.00103

**Published:** 2019-03-11

**Authors:** Xiaowan Han, Shiqiang Gong, Ni Li, Xiao Wang, Peng Liu, Yanni Xu, Xiaobo He, Wei Jiang, Shuyi Si

**Affiliations:** ^1^ NHC Key Laboratory of Biotechnology of Antibiotics, Institute of Medicinal Biotechnology, Chinese Academy of Medical Sciences and Peking Union Medical College, Beijing, China; ^2^ Department of Pharmacology, School of Pharmacy, China Medical University, Shenyang, China; ^3^ State Key Laboratory of Bioactive Substance and Function of Natural Medicines, Institute of Materia Medica, Chinese Academy of Medical Sciences, Beijing, China

**Keywords:** osteoprotegerin, receptor activator of NF-κB ligand, osteoblast, osteoclast, osteoporosis, Wnt/β-catenin signaling

## Abstract

The ratio of osteoprotegerin (OPG) to the receptor activator of NF-κB ligand (RANKL) is a key determinant in the regulation of bone metabolism. The study was performed to screen novel anti-osteoporotic drugs regulating OPG/RANKL ratio and evaluate their effect on bone metabolism. According to the screening results and *in vitro* results, we found a small molecule, E09241, significantly increased the ratio of OPG/RANKL by mainly increasing OPG expression. Our *in vitro* studies showed that E09241 increased the alkaline phosphatase (ALP) activity of mouse osteoblasts, promoted mineralization, and increased the expression of osteogenic differentiation-related genes. In addition, we observed that E09241 inhibited RANKL-induced osteoclast differentiation and reduced the expression of osteoclast differentiation-related proteins nuclear factor of activated T cells c1 (NFATc1) and matrix metalloproteinase 9 (MMP-9). More importantly, E09241 exerted therapeutic protection against bone loss in ovariectomized rats *in vivo*. This protective effect was confirmed to be achieved by inhibiting bone resorption and promoting bone formation *in vivo*. Mechanistically, E09241 regulates OPG expression through canonical Wnt/β-catenin signaling. Our findings suggest that E09241 is a promising small-molecule compound for treating osteoporosis with a dual effect on osteoblasts and osteoclasts.

## Introduction

Osteoporosis is a systemic metabolic bone disease characterized by a gradual decrease in bone mass, deterioration of the bone microstructure, and an increased risk of fracture (NIH Consensus Development Panel on Osteoporosis Prevention, Diagnosis, and Therapy, 2001). The etiology of osteoporosis is complex and an imbalance in bone remodeling is a key factor. Bone remodeling is maintained by osteoblast-regulated bone formation and osteoclast-controlled bone resorption (Karsenty, 2003). Therefore, the treatment of osteoporosis must promote bone formation or inhibit bone resorption (Rodan and Martin, 2000).

Bone remodeling is regulated by the receptor activator of NF-κB ligand (RANKL) and osteoprotegerin (OPG) (Raggatt and Partridge, 2010; Martin and Sims, 2015; Han et al., 2018). RANKL, a member of the tumor necrosis factor (TNF) superfamily, is expressed by cells that include osteoblasts and osteocytes (Kong et al., 1999). RANK, the receptor for RANKL, is found on osteoclasts and osteoclast precursors. When RANKL binds to RANK, osteoclast differentiation is activated (Dougall et al., 1999; Boyce and Xing, 2007). OPG, which is also a member of the TNF receptor superfamily, is produced by numerous cell types, including osteoblasts and bone marrow stromal cells (Yasuda et al., 1998). OPG is a soluble decoy receptor for RANKL and prevents RANKL from binding to RANK, thereby inhibiting the differentiation of osteoclasts (Quinn et al., 1998; Boyce and Xing, 2008). Osteoblasts regulate osteoclast differentiation by secreting and expressing OPG and RANKL (Goto et al., 2011). Therefore, the balance between OPG and RANKL in bone is the critical determinant in regulating bone metabolism.

In this study, we identified E09241 as an OPG/RANKL upregulator, which increases OPG expression but does not affect RANKL expression, by using an OPG/RANKL ratio high-throughput screening (HTS) system. We then explored the effect of E09241 on bone metabolism and revealed that E09241 critically affected the OPG/RANKL ratio and promoted osteoblast differentiation while inhibiting osteoclast differentiation. Furthermore, E09241 rescued rats from ovariectomy-induced bone loss. Further study revealed that E09241 regulated OPG expression through canonical Wnt/β-catenin signaling. Our findings demonstrate the potential role of E09241 in osteoporosis treatment and highlight OPG/RANKL as a promising target for osteoporosis.

## Materials and Methods

### Reagents

Alpha modification of Eagle’s medium (α-MEM), McCoy’s 5A medium, and Dulbecco’s modified eagle medium (DMEM) were purchased from Thermo Fisher Scientific (Waltham, MA, USA). Alizarin red S, ascorbic acid, and β-glycerophosphate were obtained from Sigma-Aldrich Inc. (St. Louis, MO, USA). RANKL was obtained from PeproTech (Rocky Hill, NJ, USA). All antibodies were purchased from Abcam (Cambridge, UK). Compound E09241 was obtained from the National Laboratory for Screening New Microbial Drugs, Peking Union Medical College (Beijing, China). Compound E09241 was dissolved in dimethylsulfoxide (DMSO) in all *in vitro* assays and 1‰ DMSO (final concentration) was used as a solvent control named 0 μM E09241.

### Cell Culture

MC3T3-E1 mouse calvarial preosteoblasts were obtained from the Cell Center of the Chinese Academy of Science (Shanghai, China). Human osteosarcoma cell lines U-2OS, murine monocytic cell lines RAW 264.7, and murine mesenchymal C3H10T1/2 cells were purchased from the Cell Center of Basic Medicine, Chinese Academy of Medical Sciences (Beijing, China). MC3T3-E1, U-2OS, RAW264.7, and C3H10T1/2 cells were cultured in α-MEM, McCoy’s 5A, DMEM, and MEM, respectively, with 10% FBS (Life Technologies, Carlsbad, CA, USA). Osteogenic differentiation of MC3T3-E1 cells was induced using an osteogenic induction medium containing α-MEM supplemented with 10% FBS, 50 mg/ml ascorbic acid, and 10 mM β-glycerophosphoric acid. All cells were cultured at 37°C in a 5% CO_2_ atmosphere.

### High-Throughput Screening Assay for Osteoprotegerin/RANKL Upregulator

An HTS assay was performed to identify OPG/RANKL upregulator as described previously (Gong et al., 2016). U-2OS cells were stably transfected with pGL4.17-OPGp as well as pGL4.76-RANKLp, which highly expressed both firefly and *Renilla* luciferases. After treatment by compounds for 24 h, the cells were lysed and the luciferase activity was determined by the Dual-Luciferase Reporter Assay System (Promega) with a microplate reader (PerkinElmer, Waltham, MA). The activity of a compound in the regulation of the OPG/RANKL ratio was calculated with the following formula: the regulatory activity of the OPG/RANKL ratio = the ratio of firefly to *Renilla* luciferase activities of test compound/the ratio of firefly to *Renilla* luciferase activities of vehicle control. A total of 20,000 synthetic compounds from the National Laboratory for Screening New Microbial Drugs were screened. The regulatory activity ≥150% was considered as primarily positive, and these compounds were retested in triplicate to calculate EC_50_ values.

### Alkaline Phosphatase Activity Assay

Alkaline phosphatase (ALP) activity assay was performed according to previous reports (Zhao et al., 2017). MC3T3-E1 cells were seeded in six-well plates at a cell density of 5 × 10^4^ cells/well and induced with osteogenic differentiation medium. After 12 days of induction, the cells were sonicated on ice and the supernatants were incubated with a solution containing 1.0 mg/ml p-nitrophenyl phosphate (pNPP), 0.5 mM magnesium chloride, and 1 M diethanolamine buffer at 37°C for 30 min and terminated with 3 M NaOH. The absorbance was read at 405 nm using a microplate reader (PerkinElmer). Total protein content was determined using a bicinchoninic acid (BCA) protein assay (Thermo Fisher Scientific). The ALP levels were normalized to the total protein content, and the experiments were performed in triplicate.

### Alizarin Red S Staining

MC3T3-E1 cells were seeded in six-well plates and treated with osteogenic differentiation medium for 21 days. After treatment, the cells were fixed with 4% paraformaldehyde and stained with 40 mM alizarin red S (pH 4.2, Sigma-Aldrich) at room temperature and images were taken.

### Tartrate-Resistant Acid Phosphatase Staining

RAW264.7 cells were seeded in 96-well plates at a density of 3 × 10^3^ cells/well with DMEM containing 50 ng/ml RANKL and treated with various concentrations of E09241. The cells were fixed and stained using a Leukocyte Acid Phosphatase kit (387A, Sigma-Aldrich) according to the manufacturer’s instructions. The tartrate-resistant acid phosphatase (TRAP)-positive cells with more than three nuclei were counted as osteoclasts. The osteoclasts were visualized with an optical microscope and photographed.

### Real-Time PCR Assay

Total RNA from the cells was extracted with TRIzol reagent (TransGen Biotech, Beijing, China), and reverse transcription for mRNAs was carried out with reverse transcriptional kits (TransGen Biotech). The sequences of the primers were as follows: GAPDH, 5′-AGGTCGGTGTGAACGGATTTG-3′ and 5′-GGGGTCGTTGATGGCAACA-3′; Runx2, 5′-CCGCCTCAGTGATTTAGGGC-3′ and 5′-GGGTCTGTAATCTGACTCTGTCC-3′; ALP, 5′-CCAACTCTTTTGTGCCAGAGA-3′ and 5′-GGCTACATTGGTGTTGAGCTTTT-3′; and Bglap, 5′-CAATAAGGTAGTGAACAGAC-3′ and 5′-CTTCAAGCCATACTGGTCT-3′.

### siRNA Transfection

MC3T3-E1 cells were plated in six-well plates. The cells were transfected with 50 pmol scramble siRNA (Guangzhou GenePharma Co. Ltd., Shanghai, China) or β-catenin siRNA (Guangzhou GenePharma Co. Ltd., Shanghai, China) using Lipofectamine 2000 (Invitrogen). After 6 h, the medium was exchanged with fresh medium containing E09241 and incubated for 48 h. Cells were then harvested for western blotting assays.

### Western Blot Assay

The cells were washed with PBS, and protein extracts were prepared in radio immune precipitation assay (RIPA) lysis buffer. Equal amounts of protein extracts were electrophoresed by 10% SDS-PAGE and electroblotted onto polyvinylidene difluoride membranes. The blots were blocked with 5% (w/v) skimmed milk in PBS-T buffer for 1 h and immunoblotted with primary antibodies at 4°C overnight. Then, blots were incubated with horseradish peroxidase (HRP)-conjugated secondary antibodies for 2 h at room temperature and visualized with an electrochemical luminescence reagent (ECL) detection system (Merck Millipore, Burlington, MA, USA). Data quantification and statistical analysis were conducted with ImageJ (National Institutes of Health, Bethesda, MD, USA). The membrane protein was prepared using cell membrane protein and Cytoplasmic Protein Extraction Kit (Beyotime, Jiangsu, China).

### ELISA

The levels of OPG and RANKL proteins in the cell supernatants were determined using the corresponding ELISA kit (R&D Systems, Minneapolis, MN, USA). The levels of OPG, RANKL, CTX-1, and ALP in rat serum were measured using ELISA kits (Nanjing Sen Bei Jia Biotechnology Co., Ltd., Nanjing, China).

### TCF/LEF Reporter Assays

The cells were co-transfected with pTOPflash (Upstate) or pFOPflash (Upstate), and pRL-TK *Renilla* plasmid using Lipofectamine 2000 (Invitrogen). After 24 h of transfection, the cells were treated with various concentrations of E09241. The firefly luciferase activity and *Renilla* luciferase activity were measured using the Dual-Luciferase Reporter Assay System (Promega).

### Ovariectomized Rat Osteoporosis Model

Twenty-four 7-month-old female Sprague-Dawley rats (290–310 g) were purchased from Beijing Vital River Laboratory Animal Technology Co., Ltd. (Beijing, China). Six female rats were subjected to resection of some fat tissue close to the ovaries (sham), and 18 underwent bilateral ovariectomy as previously described (Ren et al., 2016). One week after ovariectomy, the OVX rats were injected intramuscularly with 1 mg/kg dexamethasone (Sigma) twice a week for 4 weeks to induce more serious osteoporosis (Ren et al., 2016). Eighteen OVX rats were then divided randomly into three groups with six rats in each group, including the OVX group, E09241-L group, and E09241-H group, which were intragastrically administered daily with 0.3% CMC-Na, 5 mg/kg/day E09241 (in 0.3% CMC-Na), or 20 mg/kg/day E09241 (in 0.3% CMC-Na), respectively. Rats were sacrificed after 12 weeks, serum was collected, and femurs were obtained for micro-CT analysis. All animal procedures were performed in accordance with the regulations of the Institutional Animal Care and Use Committee of the Institute of Medicinal Biotechnology.

### Micro-CT

The femurs taken from rats were dissected free of soft tissue, fixed in 4% paraformaldehyde, and distal femurs were analyzed using a micro-PET/CT scanner (Inveon, Siemens, Berlin, Germany). The scanner was set at a resolution of 10 μm, with a tube voltage of 50 kV, and tube current of 400 μA. Three-dimensional reconstruction was conducted with the Inveon analysis workstation. One millimeter below the center of the epiphyseal line with 50 slices was selected as the region of interest (ROI) for the analysis, and the following bone properties were generated: bone mineral density (BMD), relative bone volume (BV/TV), trabecular number (Tb.N), trabecular thickness (Tb.Th), bone surface/bone volume (BS/BV), and trabecular separation (Tb.Sp).

### Bone Histomorphometric Analyses

The femurs were fixed in 4% paraformaldehyde, decalcified with 10% EDTA (pH 7.2), and then stained with H&E and TRAP to visualize osteoblasts and osteoclasts. The numbers of osteoblasts per bone surface (N.Ob/BS) and osteoclasts per bone surface (N.Oc/BS) were analyzed by Image Pro Plus 6.0.

### Statistical Analysis

All data are presented as mean values ± SD. Differences between values were analyzed using one-way ANOVA analysis with Tukey’s multiple comparison tests. Statistical significant was assigned to *p* values of <0.05.

## Results

### E09241 Increases Osteoprotegerin Expression *in vitro*

**Figure 1:**
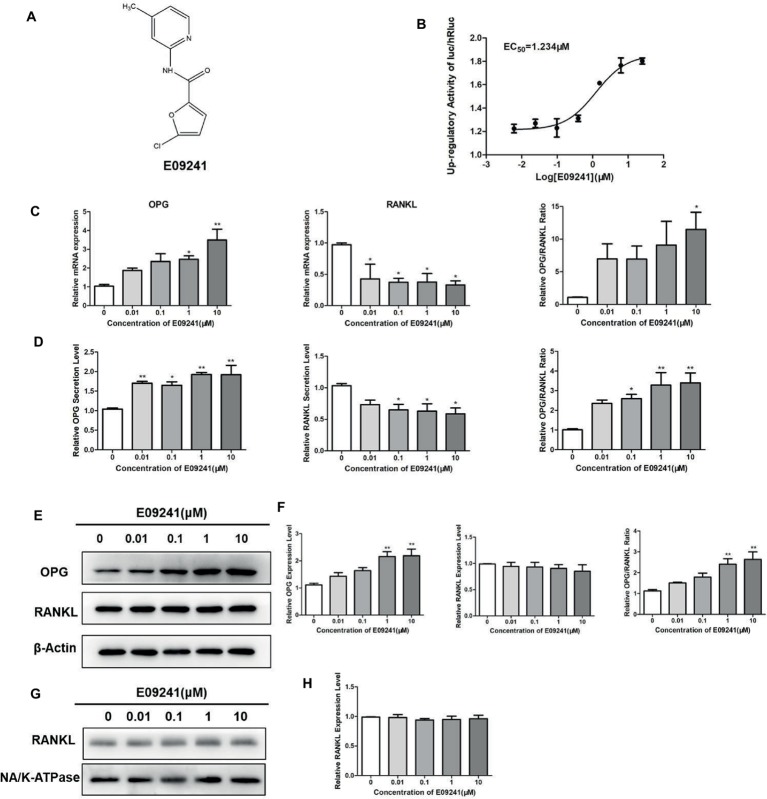
Effects of E09241 on the expression of osteoprotegerin (OPG) and receptor activator of NF-κB ligand (RANKL). **(A)** Structure of E09241. **(B)** Dose–response curve of E09241 on the OPG/RANKL ratio high-throughput screening (HTS) model. **(C)** OPG and RANKL mRNA expression and the ratio of OPG/RANKL in MC3T3-E1 cells treated with E09241 for 24 h. *p < 0.05, **p < 0.01 versus control (*n* = 3). **(D)** OPG and RANKL secretion from MC3T3-E1 cells were measured using ELISA. *p < 0.05, **p < 0.01 versus control (*n* = 3). **(E,F)** Western blot and expression levels of OPG, RANKL, and the ratio of OPG/RANKL in MC3T3-E1 cells treated with E09241 for 24 h. **p < 0.01 versus control (*n* = 3). **(G,H)** Western blot and expression levels of RANKL in the membrane of MC3T3-E1 cells. Na/K ATPase was used as a membrane protein for loading control.

E09241, the systematic name of which is 5-chloro-*N*-(4-methylpyridin-2-yl)furan-2-carboxamide (Figure 1A), was identified by HTS for upregulators of the OPG/RANKL ratio by changes in firefly and *Renilla* luciferase activities, with an EC_50_ of 1.23 μM (Figure 1B). We initially detected the expression of OPG and RANKL in MC3T3-E1 cells treated with E09241 at 0–10 μM by real-time quantitative PCR assays and western blotting. E09241 increased the mRNA levels of OPG, especially at 10 μM and decreased the mRNA levels of RANKL, thereby increasing the ratio of OPG/RANKL (Figure 1C). In addition, E09241 also increased OPG secretion in MC3T3-E1 cells (Figure 1D). Western blot results showed that E09241 significantly increased OPG protein expression level in a dose-dependent manner.

RANKL has two forms including membrane-bound form and soluble form, and the membrane-bound form RANKL is essential for osteoclastogenesis. In order to examine the effect of E09241 on different RANKL forms, RANKL levels in cells, cell membranes, and culture supernatants were detected, respectively. As shown in Figure 1D, ELISA assay showed that E09241 significantly decreased secreted RANKL in MC3T3-E1 cells, which was in accordance with the mRNA levels of RANKL (Figure 1C). Western blots results showed that E09241 had no obvious effects on the total and membrane protein levels of RANKL (Figures 1E–H).

### E09241 Promotes Osteoblast Differentiation

**Figure 2:**
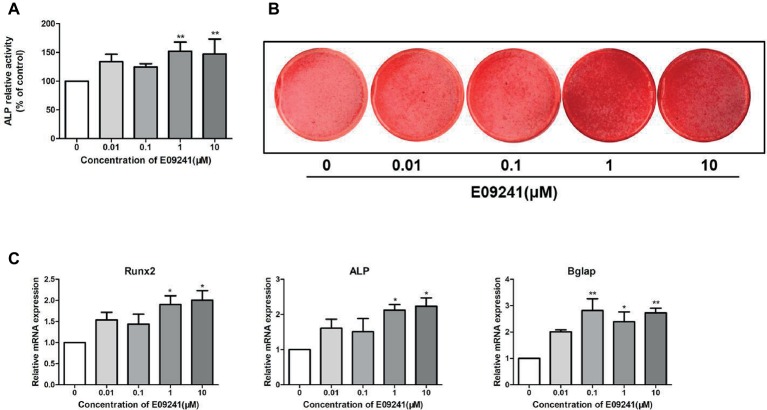
Effects of E09241 on osteoblast differentiation and osteoblast-related gene expression. **(A)** Alkaline phosphatase (ALP) activity of MC3T3-E1 cells treated with E09241 in osteogenic medium for 12 days. **p < 0.01 versus control (*n* = 3). **(B)** Alizarin red S staining of mineralization of MC3T3-E1 cells treated with E09241 in osteogenic medium for 21 days. **(C)** *Runx2*, *ALP*, and *Bglap* mRNA expression in MC3T3-E1 cells treated with E09241 for 12 days. *p < 0.05, **p < 0.01 versus control (*n* = 3). The results were normalized to the expression levels of GAPDH.

The effect of E09241 on the differentiation of osteoblast MC3T3-E1 cells was determined by an ALP activity assay and alizarin red S staining. E09241 increased the ALP activity of MC3T3-E1 cells in osteogenic induction medium with E09241, especially at 1 μM, for 12 days (Figure 2A). Moreover, MC3T3-E1 cells exhibited increased mineralized nodules after 21 days of exposure to 1–10 μM E09241 (Figure 2B).

We analyzed the effect of E09241 on the mRNA levels of osteogenic differentiation-related genes, including *Runx2*, *ALP*, and *Bglap* in MC3T3-E1 cells for 12 days. The mRNA expression of *Runx2* was increased by treatment with 1–10 μM E09241, and *ALP* mRNA expression was increased by 1–10 μM E09241 (Figure 2C). In addition, *Bglap* mRNA expression was markedly induced by treatment with 0.1–10 μM E09241.

### E09241 Inhibits RANKL-Induced Osteoclast Differentiation

**Figure 3:**
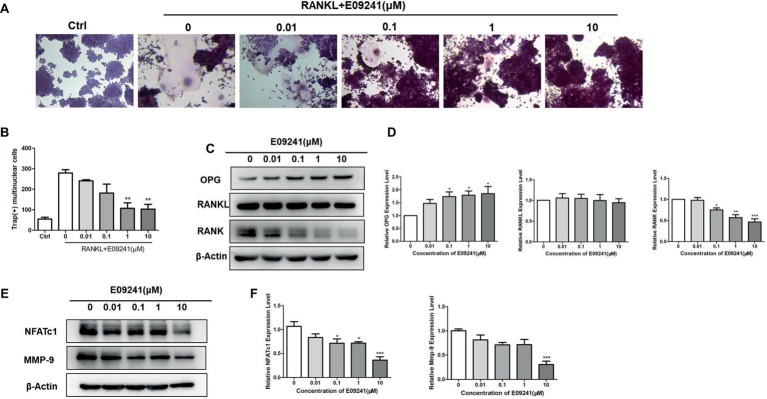
E09241 inhibits receptor activator of NF-κB ligand (RANKL)-induced osteoclast formation. **(A)** Tartrate-resistant acid phosphatase (TRAP) staining of RAW264.7 cells treated with different concentrations of E09241 in the presence of RANKL for 3 days. **(B)** Quantitative analysis of TRAP-positive multinuclear cells, for RAW264.7 cells, TRAP-positive multinucleated (nuclei > 3) cells were counted as osteoclasts. **p < 0.01 versus control (*n* = 3). **(C,D)** Western blot and expression levels of NFATc1 and MMP-9 in RAW264.7 cells treated with E09241 for 24 h. *p < 0.05, ***p < 0.001 versus control (*n* = 3). **(E,F)** Western blot and expression levels of osteoprotegerin (OPG), RANKL, and RANK in RAW264.7 cells treated with E09241 for 24 h. *p < 0.05, ***p < 0.001 versus control (*n* = 3).

To investigate whether E09241 may affect osteoclast differentiation, we treated RAW264.7 cells with 1–10 μM E09241 as well as 50 ng/ml RANKL for 3 days. Our results (Figures 3A,B) showed that treatment with E09241, especially at 1–10 μM, reduced osteoclast differentiation in RAW264.7 cells stimulated by RANKL, which indicated that E09241 also inhibited RANKL-induced osteoclast differentiation *in vitro*.

To explain the direct effect of E09241 on osteoclast differentiation, OPG, RANKL, and RANK levels in E09241-treated RAW264.7 cells were then detected. The results showed that E09241 significantly increased OPG levels and decreased RANK levels but had no obvious effect on RANKL levels in RAW264.7 cells (Figures 3C,D). Nuclear factor of activated T cells c1 (NFATc1) and matrix metalloproteinase 9 (MMP-9) are osteoclast-specific transcription factors and markers (Takayanagi, 2007; Soysa et al., 2012; Li et al., 2017). We assessed the expression of NFATc1 and MMP-9 in RAW264.7 cells stimulated by RANKL. E09241 reduced the RANKL-induced NFATc1 and MMP-9 protein expression in a dose-dependent manner (Figures 3E,F). Taken together, these results confirmed that E09241 had an inhibitory effect on osteoclast differentiation.

### E09241 Attenuates Bone Mass Loss in an OVX Osteoporosis Rat Model

**Figure 4:**
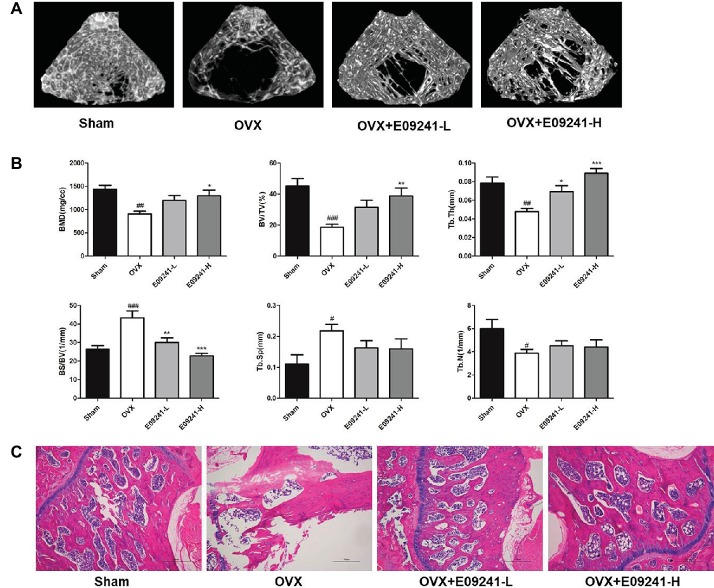
E09241 attenuates bone mass loss in an OVX osteoporosis rat model. **(A)** Representative micro-CT images of distal femurs from each group. **(B)** Trabecular bone parameters analyzed by micro-CT, including bone mineral density (BMD), BV/TV, trabecular thickness (Tb.Th), bone surface/bone volume (BS/BV), trabecular separation (Tb.Sp), and trabecular number (Tb.N). #*p* < 0.05, ##*p* < 0.01, ###*p* < 0.001 versus sham-operated group, *p < 0.05, **p < 0.01, ***p < 0.001 versus OVX group (*n* = 6). **(C)** Representative H&E staining of femurs from each group of rats at 40× magnification. Scale bars = 500 μm.

We explored the role of E09241 in an OVX osteoporosis rat model. The micro-CT images of OVX rats showed significant trabecular bone loss compared with the sham-operated group (Figure 4A), whereas treatment with E09241 for 12 weeks reduced the extent of trabecular bone loss. The bone parameters of the distal femurs were analyzed by micro-CT (Figure 4B). BMD, BV/TV, Tb.Th, and Tb.N in the OVX rats were significantly lower than those in the sham-operated rats, and BS/BV and Tb.Sp were increased. In contrast, treatment with E09241 led to a dose-dependent increase in BMD and BV/TV, particularly in Tb.Th, and a decrease in BS/BV. However, there was no obvious effect on Tb.sp and Tb.N in the E09241-L and E09241-H groups (Figure 4B). Moreover, H&E staining showed that femur structure was significantly improved in E09241-treated OVX rats compared with OVX rats, which further confirms the role of E09241 in attenuating OVX-induced bone mass loss (Figure 4C).

### E09241 Inhibited Bone Loss by Inhibiting Bone Resorption and Promoting Bone Formation

**Figure 5:**
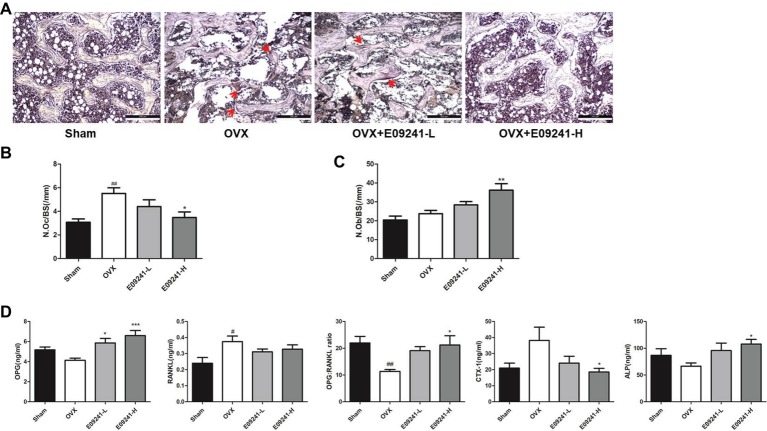
E09241 inhibited bone loss by inhibiting bone resorption and promoting bone formation. **(A)** Representative micrographs of Tartrate-resistant acid phosphatase (TRAP) staining on decalcified sections of distal femurs at 100× magnification. Osteoclasts were showed with red arrows. Scale bars = 200 μm. **(B)** The numbers of osteoclasts per bone surface (N.Oc/BS) was measured with TRAP stained sections. ##*p* < 0.01 versus sham-operated group, *p < 0.05 versus OVX group (*n* = 6). **(C)** The numbers of osteoblasts per bone surface (N.Ob/BS) was measured with H&E stained sections. **p < 0.01 versus OVX group (*n* = 6). **(D)** Serum parameters including osteoprotegerin (OPG), receptor activator of NF-κB ligand (RANKL), OPG/RANKL ratio, CTX-1, and alkaline phosphatase (ALP) were analyzed. #*p* < 0.05, ##*p* < 0.01 versus sham-operated group, *p < 0.05, ***p < 0.001 versus OVX group (*n* = 6).

To analyze how E09241 attenuated OVX-induced bone loss, we subsequently evaluated bone formation and bone resorption *in vivo* in each group. The results showed that osteoclast formation was significantly elevated in OVX rats compared to sham-operated rats (Figures 5A,B). However, in the E09241 group, the increase in osteoclast formation caused by OVX decreased in a dose-dependent manner (Figures 5A,B). Moreover, we found that the number of osteoblast was increased in OVX rats and the osteoblasts number was significantly increased in E09241-H group (Figure 5C). In addition, serum parameters were analyzed as showed in Figure 5D. We observed a decrease in serum OPG levels and a significant increase in RANKL levels in OVX rats. However, E09241 treatment significantly increased OPG levels and had no significant effect on RANKL levels, which therefore increased the OPG/RANKL ratio in rat serum. Furthermore, we found that E09241 significantly reduced the level of the bone resorption marker CTX-1 and increased the level of ALP, a marker of osteoblast function. Our results indicated that E09241 attenuated OVX-induced bone loss by inhibiting bone resorption and promoting bone formation, which was in consistent with the results of *in vitro* studies.

### E09241 Regulates Osteoprotegerin Expression Through Canonical Wnt/β-Catenin Signaling

Given that the upregulation of OPG/RANKL by E09241 is mainly through upregulation of OPG expression *in vitro* and *in vivo*, we further explored how E09241 might regulate the expression of OPG. Increasing evidence has shown that canonical Wnt/β-catenin signaling plays a significant role in regulating bone homeostasis (Krishnan et al., 2006; Kramer et al., 2010). β-Catenin is the key component of the Wnt/β-catenin signaling, and OPG is a transcriptional target for β-catenin (Glass et al., 2005); we hypothesized that E09241 might regulate OPG expression through canonical Wnt/β-catenin signaling.

**Figure 6:**
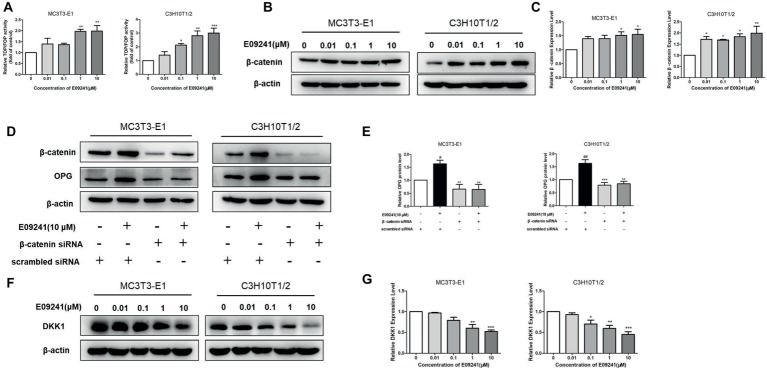
E09241 regulates osteoprotegerin (OPG) expression through canonical Wnt/β-catenin signaling. **(A)** TCF/LEF reporter activity treated with E09241 for 48 h. **p < 0.01 versus control (*n* = 3). **(B)** Western blot and **(C)** expression levels of β-catenin in MC3T3-E1 cells and C3H10T1/2 cells treated with E09241 for 24 h. *p < 0.05 versus control (*n* = 3). **(D)** MC3T3-E1 cells and C3H10T1/2 cells were transfected with scramble siRNA or β-catenin siRNA and incubated with E09241. Expression of β-catenin and OPG were determined by western blotting. **(E)** Expression levels of β-catenin and OPG were analyzed. #*p* < 0.05, ##*p* < 0.01 versus control, **p < 0.01, ***p < 0.001 versus E09241-treated group (*n* = 3). **(F)** Western blot and **(G)** expression levels of Dickkopf-related protein 1 (DKK1) in MC3T3-E1 cells and C3H10T1/2 cells treated with E09241 for 24 h. *p < 0.05, **p < 0.01, ***p < 0.001 versus control (*n* = 3).

Since β-catenin accumulates in the nucleus and activates LEF/TCF-mediated gene transcription, thereby affecting the expression of downstream target genes (He et al., 2004; Huang and He, 2008), we first examined the effect of E09241on TCF/LEF reporter activity and results showed that E09241 could increase TCF/LEF reporter activity (Figure 6A), indicating increased transcriptional activity of β-catenin. Moreover, E09241 increased β-catenin protein levels in both MC3T3-E1 cells and C3H10T1/2 cells (Figures 6B,C). We further treated MC3T3-E1 and C3H10T1/2 cells with β-catenin siRNA or scrambled siRNA to detect the expression of OPG. The results showed that knockdown of β-catenin abolished the upregulation of OPG expression induced by E09241 (Figures 6D,E).Dickkopf-related protein 1 (DKK1) is an endogenous inhibitor of the Wnt/β-catenin signaling. Furthermore, our study showed that E09241 decreased DKK1 expression in a dose-dependent manner in both MC3T3-E1 cells and C3H10T1/2 cells, which indirectly demonstrated E09241-activated canonical Wnt/β-catenin signaling (Figures 6F,G). Taken together, these results demonstrate that E09241 regulates OPG expression through Wnt/β-catenin signaling.

## Discussion

The homeostasis of bone metabolism is maintained by the balance between osteoblast bone formation and osteoclast bone resorption, and the relative levels of OPG and RANKL determine the processes of osteogenesis and osteoclastogenesis (Kong et al., 1999; Bundkirchen et al., 2018). RANKL, produced by osteoblasts, induces osteoclast differentiation by binding to its receptor, RANK. Osteoblasts also produce the soluble protein OPG, which blocks the interaction between RANKL and RANK, and thus controls the process of bone metabolism (Simonet et al., 1997; Lacey et al., 1998; Burgess et al., 1999; Lin et al., 2007). Therefore, the balance between OPG and RANKL plays a significant role in the homeostasis of bone metabolism (Anderson et al., 1997; American Society for Bone and Mineral Research President’s Committee on Nomenclature, 2000). Alternatively, an imbalance in the OPG/RANKL ratio may lead to loss of bone mass (Mountzios et al., 2007). In the present study, we used a cell-based screening method to screen small molecule that could upregulate the ratio of OPG/RANKL, which might include the following situations: screened small molecules could upregulate OPG and downregulate RANKL; upregulate OPG without affecting RANKL; do not affect OPG but downregulate RANKL; simultaneously upregulate OPG and RANKL but increase OPG more strongly; and lower OPG and RANKL but strongly reduces RANKL. In this study, E09241 significantly increased both mRNA and protein levels of OPG. Moreover, E09241 decreased mRNA levels of RANKL and secreted RANKL but had no obvious effects on the total and membrane protein levels of RANKL. RANKL has two forms including membrane-bound form and soluble form, and the membrane-bound form RANKL is essential for osteoclastogenesis. However, the RANKL protein measurements we took could not detect membrane-bound form RANKL. 125I-labeled OPG binding to cell surface RANKL assay is more suitable to detect membrane-bound form RANKL as previously reported (Yasuda et al., 1998). Thus, based on the screening results and *in vitro* results, we propose that E09241 significantly increased the ratio of OPG/RANKL by mainly increasing OPG expression.

Osteoporosis may occur when the bone resorption of osteoclasts is greater than the bone formation of osteoblasts. Thus, strategies for treating osteoporosis are based on stimulating osteoblast activity or inhibiting osteoclast activity. To investigate the effects of E09241 on osteoblasts, we examined the osteogenic differentiation of MC3T3-E1 cells after E09241 treatment. We found that E09241 promoted osteoblast differentiation, as shown by increases in both ALP activity and mineralized nodules in the osteoblasts treated with E09241. Runx-2 is a major marker of osteoblast differentiation (Ducy et al., 2000). ALP is a marker of the early stage of osteoblast differentiation and persists in early and mature osteoblasts (Aubin et al., 1995; Sims and Vrahnas, 2014). Bglap is a specific marker of late osteoblast differentiation and is expressed in matured osteoblasts (Liu et al., 2003). The expression of these differentiation markers was also significantly increased in MC3T3-E1 cells by E09241 treatment, which was attributed to the stimulatory effects of E09241on the osteoblasts. Osteoclasts, which cause bone resorption, are developed from monocyte and macrophage lineage cells (Blair and Zaidi, 2006). We showed that E09241 suppressed RANKL-induced osteoclastogenesis *in vitro* at 1–10 μM. Osteoclast formation is triggered by a sequence of RANKL-induced signaling that results in the activation of transcription factors, such as NFATc1, and *NFATc1* is a master gene for osteoclastic differentiation (Takayanagi et al., 2002; Kim and Kim, 2014). NFATc1 plays a key role in inducing the expression of genes involved in osteoclast differentiation, including MMP-9 (Song et al., 2009; Shinohara and Takayanagi, 2014). We evaluated these osteoclast markers and determined that E09241 inhibited the protein levels of NFATc1 and MMP-9.

Consistent with the *in vitro* results, our *in vivo* data in the OVX rat model demonstrated that E09241 effectively suppressed ovariectomy-induced bone loss. Based on the micro-CT image of the trabecular bone structure, our study demonstrated that both low- and high-dose E09241 treatment may prevent trabecular bone structure deterioration induced by ovariectomy. Interestingly, the bone parameters by micro-CT showed that E09241 significantly increased Tb.Th but showed no obvious effect on Tb.sp and Tb.N, indicating that E09241 mainly prevented ovariectomy-induced bone loss by thickening trabecular bone.

In view of the protective effect of E09241 on bone mass, it is necessary to clarify how E09241 contributed to inhibition of OVX-induced bone loss. We found that E09241 reduced OVX-induced increase in the number of osteoclasts *in vivo*, which suppressed bone resorption. In addition, E09241 markedly increases the number of osteoblasts *in vivo*, thereby resulting in enhanced bone formation. More importantly, we detected the levels of CTX-1 and ALP, which are markers of bone resorption and bone formation, respectively. Our results demonstrated that E09241 reduced serum CTX-1 level and increased serum ALP level, which further confirmed that E09241 attenuated OVX-induced bone loss by inhibiting bone resorption and promoting bone formation.

Wnt/β-catenin signaling plays critical roles in bone metabolism (Hill et al., 2005; Wei et al., 2011; Weivoda et al., 2016). In addition, β-catenin is a key component of Wnt/β-catenin signaling, which regulates transcription of Wnt target genes, including OPG (Glass et al., 2005). We found that E09241 amplified Wnt/β-catenin signaling and knockdown of β-catenin completely abolished the effect of E09241 on increasing OPG expression, which indicated that E09241 may regulate OPG expression by promoting Wnt/β-catenin signaling. Supportively, our study showed that E09241 decreased the expression of DKK1, which is an endogenous inhibitor of the Wnt/β-catenin signaling, and these findings suggested that E09241 might promote Wnt/β-catenin signaling by inhibiting DKK1. Studies have shown that activation of Wnt/β-catenin signaling promotes bone formation by promoting osteoblast differentiation while inhibiting bone resorption by reducing osteoclastogenesis (Bonewald and Johnson, 2008; Kramer et al., 2010). Thus, it is possible that the dual effect of E09241 on osteoblasts and osteoclasts might be mediated by activating Wnt/β-catenin pathway.

In this study, we found that E09241 enhanced osteoblast activity and decreased osteoclast formation, thereby resulting in an increase in bone volume. We proposed that mechanistically E09241 regulated OPG expression through canonical Wnt/β-catenin signaling, which upregulated the ratio of OPG/RANKL and therefore affected osteoblastic differentiation and osteoclastic differentiation. Moreover, our findings revealed that E09241 could decrease RANK levels in RAW264.7 cells, which might explain the direct effect of E09241 in RAW264.7 cells. But we do not know whether the decreased RANK level induced by E09241 is related to OPG upregulation. Thus, it is still necessary to explore whether the effect of E09241 on bone metabolism is OPG dependent, which might be conducted by detecting the effects of E09241 on bone metabolism in OPG knockout osteoblasts and *Opg* ^−/−^ mice. And, further studies will be done to obtain more insights as to the precise mechanism of E09241 on osteoblastic differentiation and osteoclastic differentiation.

In summary, the present study demonstrates that a novel small molecule E09241, which increases OPG expression but does not affect RANKL levels, protects against ovariectomy-related bone loss in rats. The effect of E09241 on OPG expression and bone metabolism may be mediated by Wnt/β-catenin signaling. Our findings suggest that E09241 is a potential compound with dual activity for preventing bone loss and osteoporosis.

## Author Contributions

XHa, SG, WJ, and SS conceived and designed the experiment. XHa, SG, NL, XW, and PL performed the experiment. XHa, YX, and XHe analyzed the data. XHa, SG, WJ, and SS drafted the manuscript. YX, WJ, and SS reviewed the manuscript.

### Conflict of Interest Statement

The authors declare that the research was conducted in the absence of any commercial or financial relationships that could be construed as a potential conflict of interest.
